# Shared Representations in Athletes: Segmenting Action Sequences From Taekwondo Reveals Implicit Agreement

**DOI:** 10.3389/fpsyg.2021.733896

**Published:** 2021-11-22

**Authors:** Waltraud Stadler, Veit S. Kraft, Roee Be’er, Joachim Hermsdörfer, Masami Ishihara

**Affiliations:** ^1^Chair of Human Movement Science, Department of Sport and Health Sciences, Technical University of Munich, Munich, Germany; ^2^Department of Human Sciences (Psychology), Tokyo Metropolitan University, Hachioji, Japan

**Keywords:** action perception, shared representations, athlete, expert, Taekwondo, action segmentation, embodied cognition, action understanding

## Abstract

How do athletes represent actions from their sport? How are these representations structured and which knowledge is shared among experts in the same discipline? To address these questions, the event segmentation task was used. Experts in Taekwondo and novices indicated how they would subjectively split videos of Taekwondo form sequences into meaningful units. In previous research, this procedure was shown to unveil the structure of internal action representations and to be affected by sensorimotor knowledge. Without specific instructions on the grain size of segmentation, experts tended to integrate over longer episodes which resulted in a lower number of single units. Moreover, in accordance with studies in figure-skating and basketball, we expected higher agreement among experts on where to place segmentation marks, i.e., boundaries. In line with this hypothesis, significantly more overlap of boundaries was found within the expert group as compared to the control group. This was observed even though the interindividual differences in the selected grain size were huge and expertise had no systematic influence here. The absence of obvious goals or objects to structure Taekwondo forms underlines the importance of shared expert knowledge. Further, experts might have benefited from sensorimotor skills which allowed to simulate the observed actions more precisely. Both aspects may explain stronger agreement among experts even in unfamiliar Taekwondo forms. These interpretations are descriptively supported by the participants’ statements about features which guided segmentation and by an overlap of the group’s agreed boundaries with those of an experienced referee. The study shows that action segmentation can be used to provide insights into structure and content of action representations specific to experts. The mechanisms underlying shared knowledge among Taekwondoists and among experts in general are discussed on the background of current theoretic frameworks.

## Introduction

Athletic performance is built on and might even extend the embodied representation of action (Beilock et al., 2008; Debarnot et al., 2014). Expert athletes specialized in specific actions form multimodal representations which are modified during years of training. The multimodal character of action representations becomes apparent, for instance, when motor knowledge is recruited in purely perceptual tasks (Aglioti et al., 2008) or when action and perception interfere (Alaerts et al., 2009; Zwickel and Prinz, 2012). Mostly for the visual domain, it has been frequently shown that athletes excel at tasks that require discriminating and predicting the actions from their own sport (Abernethy and Zawi, 2007; Smith, 2016). This is not only due to visual familiarity that results from countless times observing others during their sport but is supported by experience with the motor programs in addition (Aglioti et al., 2008; Mulligan et al., 2016). Expert athletes activated the sensorimotor system of the brain more while observing and predicting actions that they were able to perform themselves (Calvo-Merino et al., 2006; Balser et al., 2014; Apšvalka et al., 2018). Correspondingly, predicting and discriminating action can be improved by mere motor training (Casile and Giese, 2006; Aglioti et al., 2008). The interaction between perception and action might be tightened by plastic changes in the sensorimotor system which correlate with motor skill achievement (Hänggi et al., 2010; Wenger et al., 2017).

Plastic changes as a result of expertise in sport shape action representations, leading to shared knowledge among individual athletes (Schack and Mechsner, 2006; Abernethy and Zawi, 2007; Bläsing, 2014; Frank et al., 2015). Besides benefits during physical performance, shared knowledge can facilitate the perception and anticipation of action and the communication about the specific domain of expertise (Abernethy et al., 2005; Aglioti et al., 2008; Mann et al., 2010; Güldenpenning et al., 2011; Kunde et al., 2011). The topic of shared representations in sport has been addressed frequently in the context of team interaction (Rentsch and Davenport, 2006), where it was shown that teams represent not only their own but also team members’ intentions. The activation of sensorimotor functions during action perception is a potential mechanism that could underlie the understanding of teammates (Blakemore and Decety, 2001). When individuals who are on a similar level of expertise observe each other, a better match between the sensorimotor knowledge and perceived action can be assumed (Calvo-Merino et al., 2006; Schubotz, 2007; Diersch et al., 2013). However, not only sensorimotor knowledge plays a role in action understanding and interaction in sport but also semantic representations as well as strategic and normative knowledge (Rentsch and Davenport, 2006; Debarnot et al., 2014; Vesper et al., 2016). Another aspect is the level of interaction which is required to build shared representations. Besides studies in interacting teams, there are results indicating that shared representations might also exist between people who frequently engage in the same activities without interacting. For instance, it was shown that climbers had individually established the same action categories in relation to different grip shapes (Bläsing et al., 2014).

Extending this research, we studied whether indicators for shared representations can also be found in intransitive actions, i.e., when actions are not determined by the characteristics of external objects such as grips or tools. Moreover, it is an open question whether elaborate action representations would apply to unfamiliar actions from the athletes’ respective discipline. In order to measure shared representations, we used the so called “event segmentation task” (Newtson, 1973; Zacks et al., 2001; Schubotz et al., 2012) and asked Taekwondo experts to parse complex movement sequences from their sport. This task is frequently employed in the context of the “event segmentation theory” (EST) (Zacks et al., 2007) which proposes that we intuitively segment the ongoing flow of information into meaningful units or “events” in order to understand and encode them. The subjective placement and grain size of segmentation depends on top-down influence from action representations and on bottom-up sensory cues such as observed movement kinematics and top-down influence from memory (Zacks et al., 2009). Boundaries between segments are characterized by either a decrease in perceptual coherence and/or an increase in the amount of information. Several continuations are possible at boundaries which results in a transient reduction of the predictability of the forthcoming sequence (Zacks et al., 2007; Schubotz et al., 2012; Schiffer et al., 2015). For instance, in goal directed actions, individuals largely agree on placing boundaries at action goals, reflecting that sequences are less predictable after a goal has been achieved (Levine et al., 2017).

The segmentation task is suited to study the specific influence of expertise on action representations as the sensorimotor repertoire seems to determine the parsing of observed action. In patients it was shown that segmentation performance predicted impairments in activities of daily living (Bailey et al., 2013). Moreover, reduced temporal precision during segmentation was observed in participants with Parkinson’s disease (Schiffer et al., 2015). This was taken to reflect problems with movement timing known to result from dysfunctions in areas affected by the disease. That the same representations underlie segmentation and production of action is further supported by brain imaging studies. The angular gyrus in the parietal lobe and the superior frontal sulcus both associated with action selection during planning, were particularly active at boundaries between two segments (Schubotz et al., 2012). Moreover, the additional engagement of the motion sensitive temporal area (MT) suggests the attention to kinematic features at boundaries (Schubotz et al., 2012).

Beside the impact of sensorimotor impairment, also motor skill increase is reflected in action segmentation, as addressed by a few studies. Most of them involved closed-loop, mostly internally guided actions, i.e., dancing and figure skating (Bläsing, 2014; Levine et al., 2017; Di Nota et al., 2020) and recently basketball (Newberry and Bailey, 2019; Newberry et al., 2021). Different aspects were highlighted in these studies and some criteria were identified to possibly determine segmentation judgments. First, the grain size of segmentation was larger in expert dancers (Bläsing, 2014; Di Nota et al., 2020) or in amateurs after having trained the observed choreography (Bläsing, 2014). This means that amateurs marked more boundaries before they had experienced performing a dance sequence and integrated single segments into longer units after gaining motor familiarity. This effect of expertise was shown to interact with factors such as time on task (Di Nota et al., 2020) and could be changed by the explicit instruction to do fine grained segmentation (Newberry et al., 2021). A second aspect is transfer of segmentation patterns to unfamiliar actions. One study (Di Nota et al., 2020) addressed this question but found no evidence. Thus, transfer has been studied so far in one single example with Bharatanatyam dancers, an Indian dance, segmenting a ballet piece. More evidence is required here. A third aspect is inter-individual agreement about where to place boundaries in an action sequence. In videos of figure skating routines and basketball games, experts agreed on more boundaries than novices, which could indicate the use of expert knowledge (Levine et al., 2017; Newberry et al., 2021). However, in figure skating, experts and novices tended to place most boundaries at the goals of the actions or at the beginnings (Levine et al., 2017). As the authors point out, the contribution of action semantics, movement kinematics, and expert knowledge could not be clearly separated (Levine et al., 2017). Regarding basketball, the placement of boundaries could not be related to a single individual’s ongoing actions but rather to interactions within teams or with opponents.

The present study aimed at dissociating the influence of shared expert knowledge from more general aspects of action semantics and movement kinematics. Moreover, it asked whether expert knowledge generalizes such that it can be applied to unfamiliar sequences from the same domain of expertise. The Taekwondo form discipline was chosen as this sport fulfills several criteria which are crucial to address these aims. In Taekwondo, athletes can specialize in movement techniques, so called “forms” or “patterns” which are continuous sequences of complex movements derived from combat actions. They are performed individually by a single athlete. Comparable to dance, forms contain intransitive actions, which are not directed toward a goal in the environment. This allowed studying shared representations without involving any external objects which could determine action types or indicate goal achievement. In order to study knowledge transfer, experts and actions from two different Taekwondo federations, World Taekwondo (WT) and International Taekwondo Federation (ITF), were involved which allowed comparing familiar to unfamiliar actions as athletes are trained according to the techniques of one particular federation. WT has 17 and ITF has 20 different forms with increasing complexity. For instance, the first forms trained by beginners are characterized by easier transitions between movements and patterns are repeated symmetrically to four sides. Higher-level forms are mastered by experts and are more complex with less repetitions. Here we chose 12 examples, 6 from each style with different complexity. In order to assess unbiased and spontaneous agreement among athletes and to point to the preferred source of information, i.e., visuomotor or semantic, no particular grain size was instructed and the participants were encouraged to set segmentation marks subjectively in the ongoing video. This is in contrast to most earlier segmentation studies which have presented single examples of a longer action sequence several times, often under different instructions, e.g., to perform fine grained after coarse grained segmentation.

We expected that under these conditions experts spontaneously integrate over longer episodes resulting in a larger grain size, i.e., lower response frequency compared to an unexperienced control group. We further hypothesized that in contrast to the control group, Taekwondoists rely on shared expert knowledge even when observing and segmenting examples of actions from the different style for the first time. High agreement within a group leads to an accumulation of responses in particular time windows which can be described as peaks in agreed event boundaries.

## Materials and Methods

### Participants

A group of 24 Taekwondo experts was compared to a group of 29 novices. Four experts were Japanese and were tested in Japan [Department of Human Sciences (Psychology), Tokyo Metropolitan University]. Table 1 provides a detailed overview over the sport-specific expertise of both groups. The remaining 20 athletes were tested in Germany, 5 of them, who were specialized in technical form Taekwondo, at a laboratory at the Chair of Human Movement Science, Technical University of Munich and 15 members of the German national Taekwondo team [Deutsche Taekwondo Union (DTU)], specialized in sparring, were tested at the occasion of a training camp. The experts had on average 15.3 ± 6.8 years of experience and trained 13.7 ± 7.2 h per week. Members of the control group were active in their respective sport since 14.6 ± 7.3 years with 4.0 ± 1.8 weekly training hours. Most of the athletes (19) were trained according to the rules of WT, the four Japanese athletes were trained according to the ITF and one athlete was experienced in both styles. Of the control participants, 11 were tested at the lab in Japan and 18 were tested in Germany. On average, the control group had 14.6 ± 7.3 years of experience in their respective discipline and trained 4.0 ± 1.8 h per week. The participants signed an informed consent and those who came to our laboratory received allowance for travel expenses. The procedure followed the standards of the Declaration of Helsinki and was ethically approved by the Ethics Committee of Tokyo Metropolitan University (H28-69) and the vote was transferred for testing in Germany.

**TABLE 1:** Details of participant sample.

Participants	Experts Mean (SD); median; min; max	Controls Mean (SD); median; min; max
*N*	24 (4 Japan; 20 Germany)	29 (11 Japan; 18 Germany)
Gender	15 m; 9 f	15 m; 14 f
Age	23.5 (7.4); 22.0; 18; 47	25.0 (5.3); 24.5; 20; 47
		Age difference: *t*(48) = 0.86; *p* = 0.40

**Experience**	**Taekwondo**	**Other sports**

Taekwondo style	*N* = 19 WT *N* = WT + ITF *N* = 4 ITF	No experience in Taekwondo
	*N* = 15 members of German national team (DTU)	*N* = 17 game sports* N = 2 coordinative sport**
		
Experience (years)	15.3 (6.8); 16; 2.4; 31	14.6 (7.3); 17.5; 0.6; 23
Training per week (h)	13.7 (7.2);14.5; 3; 24	4.0 (1.8); 4; 1; 7.5
Duration training unit (min)	107.3 (21.1); 102.5; 90; 180	78.3 (24.9); 75;35;120
Units per week (average in last 12 months)	7.7 (4.0); 9; 2; 14	3.2 (1.4); 3; 1; 6
Participation in competitions	Currently participating = 21 No participation = 3	Currently participating = 6 No participation = 19 In the past = 4
		
Experience performing Taekwondo forms	No = 9 Yes = 13 Average 2.1 (1.5) times/week min 0.25; max 5.5 No response = 2	
Experience watching Taekwondo forms	No = 7; Yes = 15 No response = 2	

*To assess how many control participants had experience with coordinative sports, we included soccer, volleyball, basketball, and tennis but also endurance sport and shooting. **Gymnastics and Yoga.

### Stimuli

Movement sequences of the two Taekwondo styles, WT (Poomsae) and ITF (Tul) were recorded using two highspeed cameras (JVC, frame rate 250 per s) for later movement analysis and a high-resolution camcorder (Sony HDR-CX900, 1080p, 50 per s) for stimulus production. The latter camera was placed at the center and the highspeed cameras more lateral, covering the standard movement areas of 8 m × 8 m in WT and 9 m × 9 m in ITF.

**FIGURE 1:**
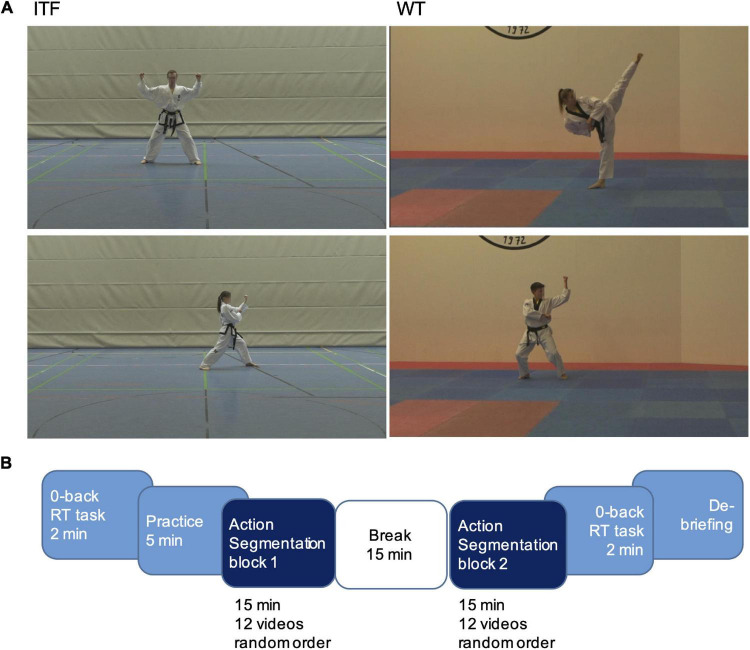
**(A)** Stimuli were 12 videos showing Taekwondo form sequences performed by two athletes experienced in ITF and two in WT Taekwondo styles. **(B)** Experimental procedure.

For each style, a male and a female athlete who were top ranked in international competitions of their federation were filmed while performing forms of lower and higher complexity. Six different Tul, number 5, 6, 7, 9, 12, and 13 were included in the experiment. The numbers 5, 7, and 13 were performed by a male athlete. The six Poomsae were, number 5, 7, 8, 9, 10, and 12 and 5, 8, and 10 were performed by a male athlete (Figure 1). In each style, the three lower numbers are easier forms, trained by pupils, and the three higher numbers are more complex which are mastered at high-level promotions. On average, an ITF clip lasted for 51 s and a WT clip for 71 s. Four additional forms, four Poomsae and four Tul, performed by male and female models were used in a practice run and one additional clip was shown as an example during the instruction. The videos were presented on notebooks with a screen diameter of 15.6 inches (33.5 cm × 19.4 cm) and a resolution of 1366 × 768. The participants were seated at a distance of 45 cm from the screen. Form this position, the height of the athletes vertically covered a visual angle between approximately 12.7° and 16.4° which varied slightly during the movement, depending on the athlete’s position in relation to the camera. The athletes’ faces were blurred to avoid that observers would recognize them or interpret gaze which might have been altered due to the proximity of cameras. The software Adobe Premiere Pro was used for video processing. For stimulus control and response recording, the software Presentation (Neurobehavioral Systems) was used.

### Task and Procedure

The 12 videos were presented in a pseudorandom order in an online version of the action segmentation task. Online means that the participants were required to mark boundaries during the ongoing video. In contrast to other versions of the segmentation task, they were not allowed to scroll forward or backward or to stop the video. This version of the task was chosen as it allows to interpret the timing of the marked boundaries in relation to the actions and to other participants.

At the start of the experiment, the participants were asked to place their right hand on the computer mouse. The instruction for the segmentation task was as follows: “Your task is to cut the action sequences into single pieces according to your own estimation. To do this task, press the mouse with your index finger whenever a unit is completed. Usually, at the same moment, a new unit is about to begin. There is no right or wrong in this task and your decisions are considered completely subjective.”

In the beginning of an experimental session, the participants signed an informed consent form, read the task instruction and watched a video of a Taekwondo form to show them which stimuli to expect. To assess individual reaction times (RTs), they performed a 0-back task in which they were required to respond as fast as possible to the appearance of an initially defined target picture. In total 50 images of different body parts were randomly presented with a stimulus duration of 500 ms and an inter-stimulus-interval of 100 ms. Among these stimuli, the target appeared 10 times (20%). The target was randomly selected out of 10 different images showing closeups of a model’s elbow, knee, feet, hands, shoulder, etc. Only responses with latencies between 100 and 1500 ms were recorded. Accuracy was recorded in addition to RT. The same response mode and device was used as in the segmentation task. This was followed by a familiarization run in which the participants practiced the action segmentation task during 5 min on four clips. All participants claimed to have understood the task and proceeded to the first experimental block in which the 12 video clips were presented in pseudo-random order with no more than 3 repetitions of the same athlete or Taekwondo style. After a break of at least 15 min, a second block of the same task but with a different order of videos was performed. To test for effects of fatigue on RT, a second run of the 0-back task was performed in the end of the session using the same stimuli and procedure as in the first run in a different order. Finally, the participants filled in a questionnaire asking about their experiences in Taekwondo and in other sports and about strategies applied in the segmentation task.

### Data Analysis

The average time between responses (TBR) was computed as a measure to compare the response frequency between the conditions. The parameter TBR was calculated for each participant and each clip by obtaining the duration between successive responses in seconds. It was measured from the first response onward, until the last and thus the times in the beginning and in the end of the videos in which the movement needs some time to start and no boundaries were marked, are not considered. For each participant, a measure of re-test reliability was obtained by correlating TBR averaged over all videos in block 1 with the average TBR in block 2. As a measure of within subject consistency, the rate of overlap (ROO) between responses in the two experimental blocks was determined by assessing the percentage of block 1 responses which were repeated in block 2. Responses were considered as repeated if they were overlapping within a time window which was individually defined for each participant. Its size was determined by the standard deviation (SD) of each individual’s RT measured in the 0-back task. As a Wilcoxon-test did not reveal a significant difference between the two runs of the 0-back task (experts: *Z* = −0.1,51, *p* = 0.13; novices: *Z* = −0.94, *p* = 0.35), the SD was computed over all trials, separately for each subject. Thus, for each response in block 2, it was controlled whether in block 1 a response was given within a time window centered at the block 2 response ± 0.25 SD. Parameter ROO represents the percentage of responses in block 2 in which a match was found in block 1.

#### Measures of Between-Subject Agreement

**FIGURE 2:**
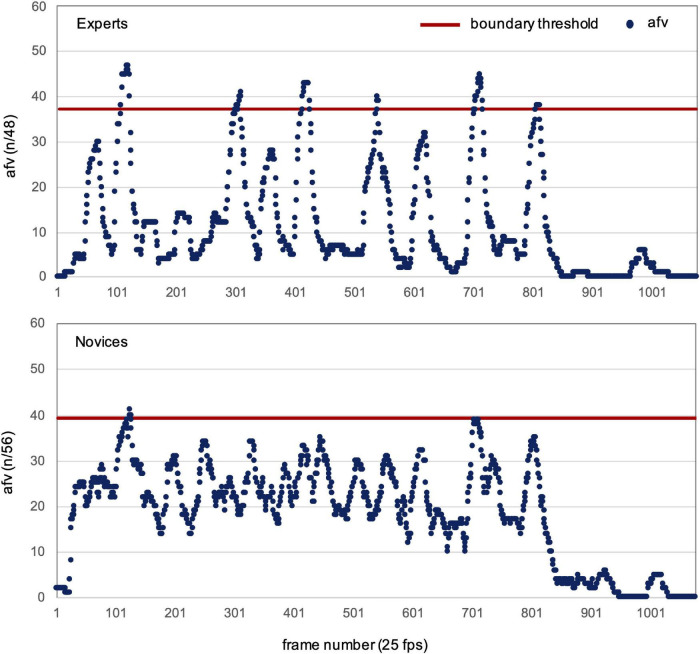
Agreed boundaries in expert and novice groups in one exemplary video (WT Poomsae #5). The added frame values (afv, blue dots) represent the number of participants responding within each bin (i.e., within 1 s prior to each video-frame). Responses of the two blocks are summarized, thus the maximal afv achievable is double the participants in each group. Boundary threshold (red line) is 2 SD above average afv. Peaks exceeding the boundary threshold are counted (n-bound) and represent within-group agreement.

The agreement about the placement of segmentation boundaries was assessed separately for each group and video. Thus, for each video, we counted how many participants within each group agreed on the same boundaries. The number of participants who responded within a time window of 1 s prior to each video frame (“bin”) was counted. This procedure was adapted from Schubotz et al. (2012) where it is described in more detail. Within the same bin, only one response of each participant was counted and thus the maximum possible number of responses was equal to the number of participants in the respective group. This resulted in the “added frame value” (afv) parameter. It contains the number of participants responding within each bin (Figure 2). In order to detect meaningful agreement, i.e., agreed boundaries (n-bound), in each video, those bins in which the afv exceeded the mean by 2 SD were identified. Means were calculated without bins with zero responses. Thus, the parameter n-bound represents above-average agreement about segmentation between participants in each group.

In the statistical analysis performed with IBM SPSS 23 we differentiated between two types of analyses.

(1) The analyses of response frequency and the response consistency were performed on values of single participants. Non-parametric tests were used since some variables were not normally distributed as tested with Shapiro–Wilk tests. Thus, for the parameters TBR and ROO, Wilcoxon tests were performed separately for experts and novices to test for differences between the styles (WT vs. ITF) and between the two experimental blocks. To test for between-group differences in TBR and ROO, Mann–Whitney *U*-tests were applied.
(2) The analyses of within group agreement on boundaries were based on the n-bound count of each single video. Thus, single videos were treated as subjects. This resulted in 12 cases, 6 ITF and 6 WT. In both styles, six videos were easy and six difficult. As each video was watched by the expert- and the control group, the group difference in n-bound was tested in a one-way repeated-measures ANOVA. Separately for each group, two multivariate ANOVAs were performed to assess the influence of (1) style and (2) difficulty on the outcome variables n-bound, average TBR and average ROO for each video.

#### Exploratory Descriptive Analyses

In addition to questions about sport specific expertise (Table 1), the participants were asked about strategies applied during the experiment in one multiple-choice question (Table 2). They could select among five different suggestions – direction change, kicks or punches, change in movement speed, predicting events in time and feeling the movement. In addition, they could indicate other criteria if applicable. In three open questions Taekwondoists were asked on experiences and thoughts during Tul/Poomsae performance and learning. Two novices and 10 athletes, all from Germany, answered the expertise questions only and did not fill in the second part due to time constraints.

**TABLE 2:** Debriefing questionnaire.

Criteria for segmentation	Experts %	Controls %
Direction change	56	70
Kicks or punches	39	74
Change in movement speed	56	59
Predicting events in time	67	19
Feeling the movement/simulation	56	15
Other criteria	22	37
	Attack and defense, connect meaningful events, or symmetric movement	Breaks, hand movement, and starting posture
Change of strategy	44	44

*Answers to the questions “Which criteria did you use for segmentation?” and “Did you change your approach during the experiment?” In the first question, multiple selections were possible and participants could optionally indicate which other criteria they used.*

The n-bound obtained within each group were compared with the boundaries defined by an expert referee who is a member of the Poomsae Referee Committee WCTU and world champion (referred to as “T4” hereafter). He was also active in training for 9 h per week. The bins in which the responses were given by T4 were graphically highlighted and superimposed on the agreed boundary graphs of the expert group (without T4) and of the control group (Figure 5). The overlaps were visually inspected.

Further, a video-based movement analysis was performed using the software SIMI Motion for a descriptive comparison between kinematics and boundaries. Synchronous recordings of three cameras were imported and prepared for 3D analysis. Six body parts, head, left and right hand, pelvis, left and right foot were marked in the first video frame and were tracked semi-automatically (with manual corrections) for the whole duration of the action sequence which corresponded to the duration of the same video in the segmentation experiment. From marker displacement in *x*, *y*, and *z*, integrated acceleration measures were obtained for all 6 markers. In order to test whether maxima in acceleration coincide with boundary marks, the acceleration profiles were treated in the same way as the afv profiles of the two groups. Thus, those periods which exceeded average acceleration by 2 SD in the positive or negative direction were marked. These profiles were graphically superimposed onto the agreed boundaries of both groups (Figure 6).

## Results

### Response Frequency and Response Consistency

No difference between the groups was found in RT in the 0-back task on images of body parts (*Z* = −0.44, *p* = 0.66). Experts needed on average 384.8 ± 59.7 ms and novices 385.71 ± 37.1 ms to respond to the presentation of the target image. Participants tested in Japan had a significantly higher mean RT (405.35 ± 43.53 ms) than those tested in Germany (377.9 ± 48.43 ms), Mann–Whitney *U*-test *p* = 0.041, which resulted from 10 German participants with RTs between 276 and 346 ms while for all 14 participants from Tokyo an RT of at least 357 ms or higher was recorded. In all other parameters, no significant laboratory effects were found. In the segmentation task, on average 15.1 ± 10.0 (median = 10.10) responses were given per video which can be expressed in a response rate of 0.25 ± 0.17 (median = 0.17) responses per second. A huge variation between the participants was observed, ranging from 5.1 to 35.9 responses per video.

**FIGURE 3:**
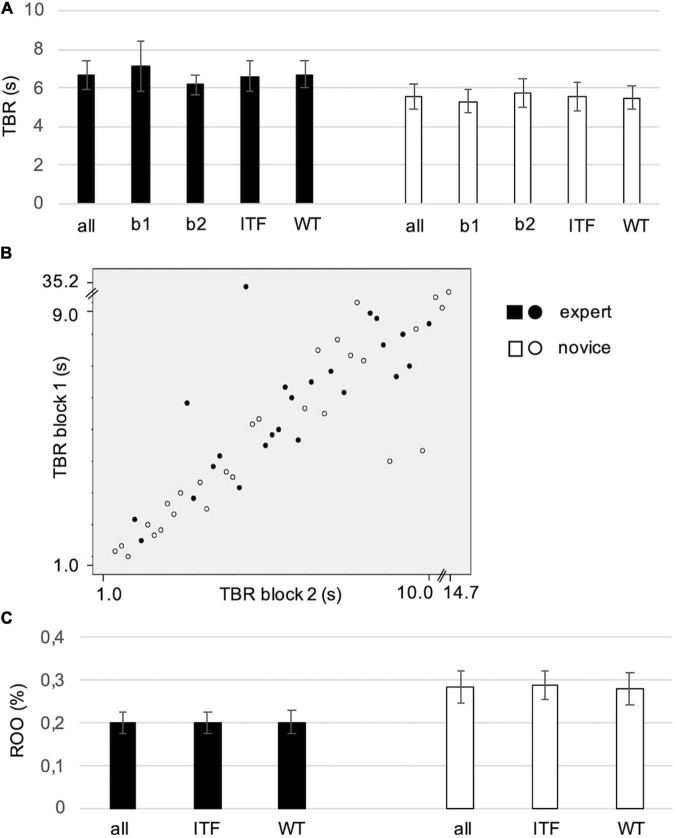
**(A)** Average time between responses (TBR) in seconds (s) for experts and novices in experimental block 1 (b1) and block 2 (b2) and for two styles (ITF, WT). Error bars indicate SE. **(B)** Correlations of TBR in block 1 and block 2 for the two groups. Scales are adjusted to values and extreme values represent two outliers (expert in block 1: 35.2 s and novice in block 2: 14.7 s). **(C)** Average response overlap (ROO) expresses the rate of block 1 responses repeated in the same time window in block 2. Error bars indicate SE.

For the statistical assessment of the effects of group, experimental block and style on the response frequency, the parameter TBR was used as outcome variable. On average, TBR was 6.64 ± 3.62 s in experts (median = 6.3; range 1.7–20 s) and 5.51 ± 3.42 s in novices (median = 5.3; range 1.7–14.1 s). Neither expertise (*Z* = −1.19, *p* = 0.23), nor style (*Z* = −0.29, *p* = 0.77) or the experimental block (*Z* = −1.21, *p* = 0.23) had a significant effect on TBR (Figure 3A). Also, when computed separately within the groups, no significant effects were found for block or style (*p* > 0.27).

To conclude, TBR reflected very different individual approaches but no systematic effects of group, experimental block and style. Correlations between TBR in block 1 and block 2 over the whole sample (*r* = 0.86, *p* = 0.001, *N* = 52) and within each group (experts: *r* = 0.76, *p* = 0.001, *N* = 24; novices: *r* = 0.92, *p* = 0.001, *N* = 28) pointed to a high re-test reliability regarding the individually selected grain size (Figure 3B). Thus, the participants kept their initial segmentation grain size.

In order to measure within subject consistency in the placement of event boundaries, the rate of overlap (ROO) was computed. No differences between groups (*Z* = −1.10, *p* = 0.27) or styles (*Z* = −0.36, *p* = 0.72) were found and the comparisons between the styles within each group were not significant (*p* > 0.22) (Figure 3C).

### Within-Group Agreement on Boundaries

**FIGURE 4:**
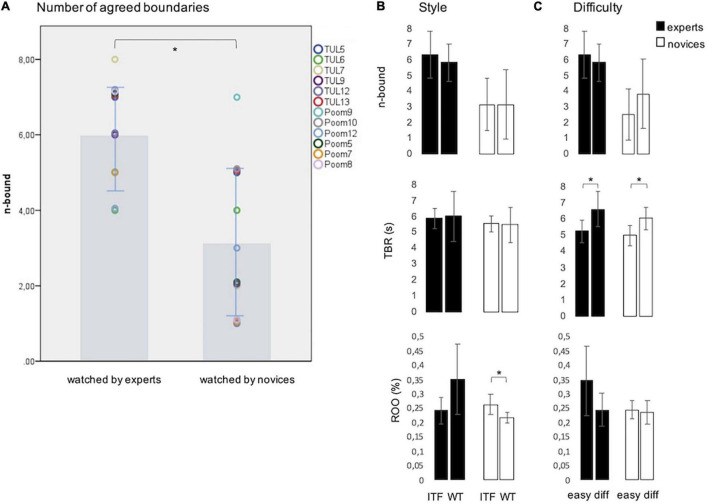
**(A)** Experts agreed on a higher number of boundaries (n-bound) than novices. Colored points represent n-bound in each of the 12 single videos watched by experts and novices. **(B)** Style (ITF vs. WT) had no effect on n-bound and on average time between responses (TBR). In novices, response overlap was significantly higher in ITF than in WT actions. The high agreement in WT in experts was not significant. **(C)** The difficulty (in terms of complexity) of the action sequences only affected TBR significantly. In both groups more time passed between boundaries in difficult sequences, i.e., the grain size of segmentation increased. In all graphs, bars show mean and error bars SD. *p < 0.05.

For each video, the parameter n-bound was determined which indicates how many peaks surpassed the threshold for within group agreement (Figure 2). To assess the effect of expertise on n-bound, the 12 videos watched by experts were compared to the same videos watched by the control group in a one-way ANOVA. As hypothesized, n-bound was significantly higher in the expert group (6.1 ± 1.31) than in the control group (3.17 ± 2.0) [*F*(1,11) = 16.29, *p* < 0.002, $\eta_{\text{p}}^{2}=0.6$] (Figure 4).

Separately for each group, two multivariate analyses of variance were performed to study the influence of either style or difficulty of the action sequence on n-bound. In order to additionally test for effects of these factors on response frequency and consistency, average TBR and ROO obtained for each video were included as additional outcome variables. In both groups, style neither had an effect on n-bound nor on TBR (*F* < 0.41, *p* > 0.54, $\eta_{\text{p}}^{2}<0.04$) but on ROO it had a marginal effect in the expert group [*F*(1,10) < 4.10, *p* > 0.071, $\eta_{\text{p}}^{2}<0.29$] and was significant in the control group [*F*(1,10) = 7.38, *p* = 0.022, $\eta_{\text{p}}^{2}=0.43$]. While experts tended to be particularly consistent in WT actions (WT: response overlap in 34.7 ± 12.1% vs. ITF: 24.2 ± 4.6%), novices were more consistent in ITF (ITF: response overlap in 26.2 ± 3.5% vs. WT: 21.7 ± 1.9%) (Figure 4B).

Difficulty did not affect n-bound and ROO in both groups [*F*(1,10) < 3.5, *p* > 0.09, $\eta_{\text{p}}^{2}<0.26$] but had a significant effect on TBR in experts [*F*(1,10) = 6.44, *p* = 0.03, $\eta_{\text{p}}^{2}=0.39$] and in novices [*F*(1,10) = 8.02, *p* = 0.018, $\eta_{\text{p}}^{2}=0.45$]. In both groups, TBR was shorter in easy sequences (experts: 5.26 ± 0.71 s; novices: 4.99 ± 0.62 s) than in difficult ones (experts: 6.62 ± 1.12 s; novices: 6.04 ± 0.67 s) (Figure 4C).

In order to address the question of transfer to unfamiliar actions, n-bound was computed for the 19 experts with WT background alone. As in the comparisons above, first, the difference to the control group was analyzed in a one-way ANOVA which resulted in a significantly higher number of n-bound in the group of WT athletes [*F*(1, 11) = 61.89, *p* = 0.001, $\eta_{\text{p}}^{2}=0.85$]. Comparing their agreed boundaries in the familiar WT videos to the unfamiliar ITF style revealed no difference between the two styles [*F*(1, 10) = 0.26, *p* = 0.62, $\eta_{\text{p}}^{2}=0.026$]. This indicates equal levels of agreement in familiar WT and unfamiliar ITF actions.

### Exploratory Descriptive Analyses for Qualitative Inspection

#### Questionnaire

In additional open questions, the participants specified which other strategies they used in the task and which general observations they made. As Table 2 shows, the control group based the segmentation more on bottom-up information. However, their open answers indicate that recurring patterns, such as the symmetric repetition of elements, were occasionally recognized by some participants. Seventeen percent of the control participants reported that they noticed that elements were repeated and that they integrated over longer sequences later in the experiment. Regarding strategy changes, the control group mostly indicated to have changed between the features listed in the questionnaire (Table 2). Athletes described the strategy of connecting elements more often than novices and in their reports the word “meaningful” was used several times, i.e., to have connected elements to longer meaningful segments. This could point to the employment of top-down semantic knowledge and is underlined by the use of prediction in experts. Trying to feel the movement was selected more often by experts and employs mechanisms of action simulation and imagery. Approximately half of the experts were confident with their strategy only for the familiar sequences. For the unfamiliar style, one expert reported to have “memorized the whole movement and then tried to get into the details.” The same strategy was reported by one control participant.

#### Comparison With Judgments of Expert Referee

**FIGURE 5:**
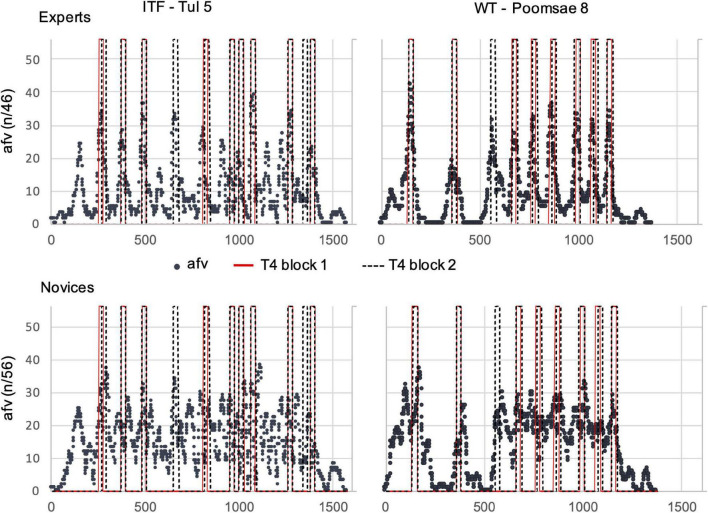
Agreement of within-group responses with boundaries marked by an experienced referee (T4). The added frame values (afv) represent the number of participants responding within each bin (i.e., 1 s prior to each video-frame). Responses of the two blocks are summarized, thus the maximal afv achievable is double the participants in each group (expert group without T4). Red lines represent the boundaries of T4 in block 1, dashed black lines are boundaries from block 2. The exact times of the responses of T4 are at the line marking the end of the 1 s bin during which they are counted (see computation of afv). Two actions, TUL #5 and Poomsae #8, are shown. The complete set of figures for all videos and both groups is provided in Supplementary Figure 1. In addition, the two videos with concurrent afv of both groups are provided as Supplementary Material.

Comparing the responses of the referee T4 between the two blocks, it is obvious that they frequently fell into the same bins (Figure 5). Thus, the boundaries marked by T4 largely corresponded between the two blocks. In experts, afv-peaks overlapped more often and more precisely with the responses of T4 than the afv maxima in novices. This precise overlap points to prediction of event boundaries in experts (Schiffer et al., 2015). This was particularly clear in WT sequences of low complexity, where afv peaks in experts are steeper. Notably, 67% of the experts indicated in the questionnaire to have predicted events in time, in contrast to 19% in the control group. For some of the more complex, higher level sequences, the agreement with T4 tended to be reduced. Maxima of novices were sometimes observed in the vicinity of the boundaries marked by T4 but were less well overlapping. This could point to reactive instead of anticipatory boundary detection. However, this speculation requires further evidence.

Further, the comparison with T4 allows some conclusions on the segmentation criteria the experts agreed upon. At the occasion of debriefing T4 indicated that besides using motor simulation he relied on guidelines and regulations of the world federation. Further, he described to segment the sequences in smaller units also during learning. This corresponded to the answers of other athletes.

The complete set of figures for all forms and both groups is provided in Supplementary Figure 1. The Supplementary Videos allow to observe the relation between agreed boundaries and the movement sequence more directly in a dynamic display. The videos show an animated mark moving through the afv-graphs in synchrony with the movement video. The same videos as those Figure 5 refers to are shown.

#### Comparison With Movement Kinematics

**FIGURE 6:**
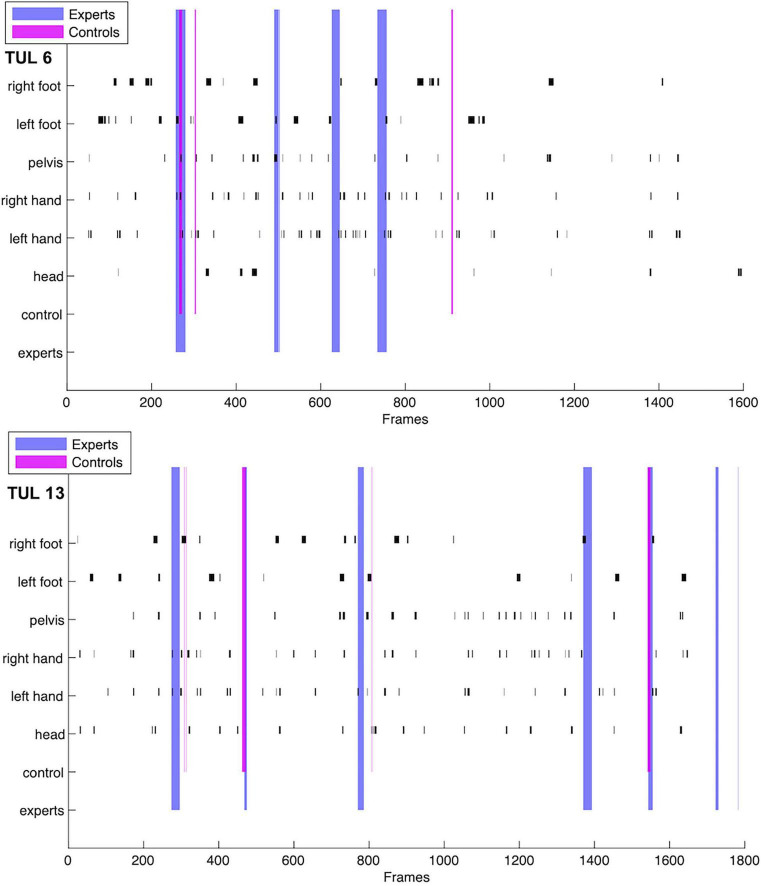
Acceleration maxima (black bars) as obtained for the whole video duration of two ITF sequences (Tul #6 and #13) are shown for six different body parts. Colored lines represent agreed boundaries in the same videos for the two groups (blue – experts; pink – novices, i.e., “controls”). Line width corresponds to length of period in which values were above the mean (i.e., agreed boundaries, n-bound).

Taekwondo form patterns are complex full-body movements for which different body parts are relevant at different times and likely have varying influence on action segmentation over the course of the sequence. To account for this complexity, we chose a procedure in which we selected maxima in acceleration/deceleration using the same approach as for determining agreement between participants. Periods in which values exceeded the mean by 2 SD were marked for each tracked body part separately. For a descriptive comparison, these time windows were graphically overlayed with intervals in which afv was above threshold in the experts and in the novice group (Figure 6). Visual inspection revealed that experts’ boundaries were occasionally aligned with acceleration maxima or minima of the limbs. As novices had less boundaries there are less occasions for such coincidences and a quantitative comparison between the two groups is unreasonable.

## Discussion

The present study aimed to shed lights on shared action representations in Taekwondo experts. Videos showing complex movement sequences of two Taekwondo styles, ITF and WT were presented to participants who were experts in one of these styles or had no experience in martial arts. They provided judgments about the structure of the action sequences by marking boundaries between meaningful units in the observed actions. We measured the within-group agreement on where to mark boundaries and hypothesized that during segmentation experts refer to shared knowledge, resulting in a higher number of agreed boundaries (n-bound). The results confirmed this hypothesis, showing that the group of experts agreed on significantly more boundaries than the novices (Figure 4). Remarkably, the experts achieved this level of agreement intuitively, without any explicit instructions about which grain size to apply or which key features they should attend to. As a consequence, there was a remarkable variation in the response frequency in both groups. The number of agreed boundaries was unaffected by Taekwondo style or the complexity of the observed sequences. We assume that different aspects of action representations have influenced segmentation behavior. Besides visuomotor cues in the observed movement, the agreement among experts seems to be based on shared representations about the function of the movement elements and meaningful combinations. The relative roles of these different factors in the context of the segmentation of Taekwondo sequences are discussed.

Within the experimental procedure, several measures were applied to control for confounding effects resulting from basic differences in task understanding, response behavior and strategies between the groups. This included between- and within-subject comparisons of RT in the 0-back control-task and the assessment of intra-individual consistency, represented by the parameter response overlap (ROO). No differences were found between the experts and the control group in these measures. Longer RT in the 0-back control task observed in participants tested in Japan could result from the different equipment used or from different strategies in this task. We found no evidence that this affected other measures.

### Grain Size, Observed Movement Kinematics, and Semantic Representations

Zacks et al. (2009) found that participants were guided by the kinematics of arm movements in the context of object manipulation more when they were instructed to do fine grained segmentation. Thus, the grain size of segmentation could be taken as an indicator for the use of visuomotor cues. When segmenting a modern dance piece, novices used smaller grain sizes which increased after amateurs had learned to dance the sequence (Bläsing, 2014). Some members of our control group reported a similar change in strategy with longer time spent on task. Employing videos of basketball games, a recent study (Newberry et al., 2021) found that experts detected more boundaries than novices when they were asked to do fined grained segmentation. This was attributed to their increased visual sensitivity for details (Newberry et al., 2021). However, without instructions that request a particular grain size, we expected experts to mark less boundaries, reflecting their use of semantic knowledge to integrate over longer episodes. In contrast, smaller grain size applied by novices should reflect their use of visuomotor cues. Contrary to these expectations, the grain size did not differ between experts and novices, as expressed in a similar average TBR.

At first glance, the similar average grain size might be taken to suggest that both groups relied on similar representations to segment Taekwondo forms. The observation that experts’ boundaries coincided more often speaks against this conclusion. This occurred despite the interindividual variability in TBR was high in both groups and although the average response frequency in experts was not different from that of novices. According to the questionnaire, experts relied on different information than novices (Table 2). These different sources are outlined in the following.

In both groups, the response frequency may have been influenced by the inherent structure of Taekwondo forms. In daily actions and also in dance or figure skating which were used in other studies on effects of expertise (Bläsing, 2014; Levine et al., 2017; Di Nota et al., 2020), a comparable structure most likely was not present. Taekwondo sequences consist of accentuated movements of the limbs which represent offensive and defensive techniques, i.e., kicks and blocks toward an imagined opponent. Single elements are frequently separated by changes in movement speed and direction or a change of the performing limb. Further, especially in Poomsae or Tul of lower complexity, the same patterns (i.e., combinations of kicks and blocks) are performed symmetrically to all 4 directions (lateral, forward, and backward). These features establish a temporal structure which could have driven segmentation in both groups, herewith determining the average TBR. In the questionnaire, participants in the control group indicated to have attended to low-level features like tempo, direction changes, and single movements. They used these criteria and combinations of them unsystematically. Some participants reported to have been driven by the rhythmic structure which was specific to the ITF sequences (see Supplementary Videos). Attention to the temporal structure of the movement could be reflected in the higher rate of response overlap in ITF observed in novices (Figure 4). Experts, in contrast tended to use event prediction and embodied simulation as they imagined how it feels to perform the action.

Semantic representations guided segmentation especially in experts in addition to the sequence structure and to visuomotor cues. As earlier findings on the role of action semantics for segmentation indicate (Levine et al., 2017), mainly experts but also non-experts attend to semantic information such as goal achievement. Here, basic motorcognitive principles such as the preference for action effect coding (Koch et al., 2004; Shin et al., 2010) could be expressed. Beyond basic principles – which special knowledge did Taekwondoists access in addition? The questionnaire pointed to the formation of clusters of elements by experts, comparable to a study by Bläsing (2014). Experts described to rely on higher level semantic representations about meaningful combinations of actions. For instance, knowing that an offense needs to be followed by a defensive movement, experts rarely separated these into different segments. Novices, in contrast, did not seem to adhere to such rules. Although occasionally their marks overlapped with those of experts and even though no differences in grain size were found, the higher n-bound indicates that experts placed boundaries in a more consistent way. The agreement with the responses of T4 is in line with this interpretation and also points to the use of normative criteria. As T4 indicated in the questionnaire, besides employing imagery, he thought of official guidelines for form practice during segmentation. This was not mentioned by any other participant but could have influenced their judgment implicitly. For instance, they would learn about normative criteria during training, such as how high the leg should be raised or whether it should be fully stretched. Another aspect to consider is that T4 and other athletes mentioned to split the sequences into smaller units when they learn them. It is possible, that, at least for familiar sequences, the agreement among athletes is supported by more or less standardized instructions on how to split, i.e., segment the forms.

Generally, temporal alignment reflects prediction of the dynamic action sequence (Schiffer et al., 2015) and likely expert knowledge was beneficial here. For responses to add up in the agreed-boundary-count, it is necessary that individuals time their responses precisely to these segment boarders. Predictive mechanisms were also shown to play a role for the identification of boundaries, as these are characterized as moments when prediction error is high (Eisenberg et al., 2018). These two functions point to predictions on different hierarchical levels of action representation. The temporal alignment of the responses with the observed dynamics might involve sensorimotor processes. The computation of prediction error can also cover larger time spans and may result from semantic action representations. For instance, referring to clustering in experts, prediction error might have been relatively low at the completion of an offensive movement, as they knew that it would be followed by a block. In contrast, in order to place a mark at the end of a leg extension movement, its timing could have been predicted on the basis of sensorimotor expertise. Kinematic and semantic features also coincide, for instance an attack-block combination ends with a decrease in movement speed. This might have caused similar TBR ranges is both groups, even though experts were guided by higher-level semantic and novices by lower-level kinematic features.

Our preliminary analysis in which we overlayed agreed boundaries with the acceleration patterns in six body parts (Figure 6) was intended to indicate whether boundaries overlap with the acceleration or deceleration, i.e., kindematics, of arms and legs differentially in both groups. Although it demonstrates descriptively that boundaries and acceleration peaks occasionally coincide, it is not suited for quantitative assessments of group or effector differences. Comparing between groups would be problematic as the frequency of overlaps would be confounded with the lower number of n-bound in novices. An additional difficulty for such an analysis was the complexity of the full body movement, as we outline in the limitations at the end of the discussion.

### Role of Familiarity of the Action Sequence

All experts were routined in performing the movement elements (e.g., kicks with hands and feet) but they weren’t equally familiar to all action sequences. Moreover, the ITF style was unfamiliar to 19 out of 24 participants. Despite the novelty of half of the sequences, the number of agreed boundaries (n-bound) was equal in both styles and also the level of difficulty had no effect on n-bound in the expert group. Comparing between familiar and unfamiliar sequences in WT athletes only confirmed these results. This points to a transfer of event segmentation to unknown action sequences, at least for this particular type of actions. The findings are in line with a study by Abernethy et al. (2005) who tested the performance in a pattern recall task based on videos of game sequences in experts from three different ball-sports and found that superior performance was to some extent transferred to different ball-sport disciplines (Abernethy et al., 2005). Similarly, studies that employ videos of everyday actions (Zacks et al., 2001; Schubotz et al., 2012) include actions which the observers have not practiced in the same sequences and in a similar context before. Together these results suggest that transfer to unfamiliar sequences is possible as long as these match to the motor repertoire of the observers and are based on known semantic principles. This includes basic principles such as the sensitivity to action goals (Levine et al., 2017). When WT Taekwondo experts segmented unfamiliar ITF forms, the observed actions matched their motor repertoire and, moreover, these known movements were composed according to familiar rules. Conversely, the reason why segmentation was not transferred in dancers who segmented unfamiliar ballet choreographies (Di Nota et al., 2020) could have been the lack of the special motor skills and knowledge of sequence structure required in ballet.

### Shared Representations Among Athletes

The generation and structure of shared representations can be explained on the basis of different theoretical frameworks. One explanation is derived from schema theory. With respect to sensorimotor functions, a schema is conceived as the relationship between (1) external conditions, (2) the motor program, (3) the sensory consequences, and (4) the outcomes of the movement (Schmidt, 1975). Repetition during training is thought to strengthen this relationship with the effect that motor programs match expected sensory consequences with increasing precision. Schemata are thought to influence perception and the recall of events. Representations shared among experts might consist of so called “scripts” which are particular schemata describing predictable and frequent action sequences (Rentsch and Davenport, 2006). Different domains in sport vary with regards to which and how many agreed-upon scripts are available or even needed (Rentsch and Davenport, 2006). The technical form discipline of Taekwondo is an example where scripts could be useful due to the stable sequential structure. For the level of agreement Taekwondoists achieved in the present study, it may have been sufficient to rely on scripts with basic rules for the combination of elements. This is derived from the observation that the participants had no experience with at least half of the observed sequences.

A second explanation is based on the cognitive action architecture approach (CAA-A). It describes action representations as integrated networks of movement elements called “basic action concepts” (BACs) (Schack, 2004). Integrating ideas from schema- and ideomotor theories it suggests that BACs are cognitive sets which link representational structures with motor performance, i.e., movements and associated perceptual effects or action goals. In this view, internal action representations are hierarchical tree-like taxonomies of BACs. Motor skill learning re-organizes hierarchies by changing the relations and clusters of BACs (Frank et al., 2015). It was repeatedly shown for experts from different sports, that the representational structure follows a distinct hierarchy which was largely similar between individuals and was in accordance with the functional phases of actions (Schack and Mechsner, 2006; Frank et al., 2015). Comparable to what is found in action segmentation, these studies suggested that in novices interindividual variability is higher and clustering of BACs does not seem to reflect a meaningful organization.

On the background of a dynamic systems approach, a recent review highlighted interpersonal synergies in combat sports (Krabben et al., 2019), suggesting that “a joint perception-action system emerges” (Krabben et al., 2019) where “the perception and action of two individuals are mutually constrained and coupled.” According to this concept, the participants in the Taekwondo group of the present study were trained in forming such dynamic synergies with another person, as the majority of them were actively competing in the combat discipline. During a fight, they need to continuously adapt their behavior to that of the opponent. Thus, they are strongly relying on observing with all their senses and predicting their opponents in order to identify advantageous moments to score. About half of the experts practiced forms. Also specialists in the technical form discipline of Taekwondo described to picture an imagined opponent during sequence performance. Irrespective of the specialization of the athletes, experience in representing the opponent might have been beneficial for the agreement in action segmentation. Research on joint action points to a similar direction, proposing that interacting persons need to represent what they can do together (Sebanz et al., 2006; Vesper et al., 2016). These authors highlighted the requirement of representing the interaction itself which goes beyond shared representations of an individual’s motor skills or the task set.

Finally, shared representations can be explained from an embodiment perspective. This perspective departs from the view that perception and cognition are grounded in bodily states and sensorimotor processes (Barsalou, 2008) and consequently cannot be thought separately from these. Motor theories of action understanding share this perspective. They assume that the same sensorimotor mechanisms underlie the production and the perception of action (Blakemore and Decety, 2001; Jeannerod, 2001; Wilson and Knoblich, 2005), a notion which has received both behavioral and neural support. Action representations interact with a number of cognitive and perceptual tasks (Rosenbaum et al., 2012). The activation of sensorimotor brain areas during the observation of others’ actions, as found in numerous studies (Hardwick et al., 2018), was conceived as “motor resonance” (Uithol et al., 2011). Through this mechanism, shared representations from similar sensorimotor experiences could be activated during observation. This builds a direct link between observed action and the observer’s action representations, so called “direct matching” for action understanding. With respect to expertise, direct matching requires some degree of correspondence between the observed action and the observer’s repertoire (Wilson, 2001). Support for this requirement was provided from brain imaging studies showing stronger activation in sensorimotor areas of movement experts who observed actions from their domain of expertise (Calvo-Merino et al., 2006; Balser et al., 2014; Cacioppo et al., 2014; Gardner et al., 2015; Apšvalka et al., 2018). Moreover, while observing and predicting skilled actions, experts activated brain representations of the muscles crucial for motor performance (Aglioti et al., 2008). Participants were better at discriminating complex actions when they had learned how to perform them before (Casile and Giese, 2006). Thus, plastic changes that occur in the brain during motor skill learning (Wenger et al., 2017) might change brain processes not only for motor performance but also for perceptual and cognitive tasks (Beilock et al., 2008; Kirsch et al., 2018). In the present study, sensorimotor knowledge achieved through practicing Taekwondo may have contributed to predicting boundaries through an insight into the dynamics and biomechanical constraints. Indeed, about half of the participants from the expert group reported having imagined how it feels to perform the movement (Table 2). At this level of action representations, motor resonance presents a framework explaining how shared representations are embodied. In the future, it will be interesting to discuss how embodiment, social embeddedness (Marsh et al., 2009) or a common task or goal (Vesper et al., 2016) can explain shared representations within teams or individual experts in sport at different representational levels.

### Limitations and Outlook

This study certainly has a couple of limitations and can be considered preliminary from many perspectives. First, it cannot quantify the relative contributions of the different aspects of action representations, i.e., movement kinematics, basic and expert semantics. The qualitative results provide some insights but the unsystematic and exploratory character of the employed methods has to be admitted. The overlay of the group-wise n-bound with the responses of the expert referee (Figure 5) is a descriptive approximation. As long as the criteria behind each of the referees marks are not known exactly, the underlying thoughts can only be derived from more general responses in the questionnaire and, thus, caution is required when interpreting this comparison. Second, we explicitly chose a design which allowed studying implicit agreement but which was not suited to assess the hierarchical organization of action representations (Schack, 2004; Zacks et al., 2009). Third, to test the transfer of segmentation behavior to an unfamiliar style of Taekwondo more systematically, it would have made sense to differentiate between experts in ITF and WT among Taekwondoists to compare the effect of familiarity in a complete cross-over design. Unfortunately, it was not possible to recruit enough ITF athletes for such a comparison. Fourth, as an approach to assess how boundaries are related to movement kinematics, cross-correlations between segmentation patterns and limb acceleration could be computed (Zacks et al., 2009). Ideally, this could reveal temporal relations between the signals to study, for instance, the anticipation of events. This was not doable in the present study, due to the complexity of the movements. A tracked limb was not continuously in use during an entire sequence but different body parts were engaged in alternation. For example, a powerful and fast attack was performed with one leg while the arms were relatively still. This was followed by a fast extension of an arm moving the hand to the front with the feet standing sill and stable on the ground. Consequently, segmentation could have been initially determined by velocity changes of the leg but later in the sequence the contribution of the leg was minimal which would cancel out in a correlation analysis. Future studies might find a way to address the coupling between boundaries and kinematic patterns in complex movement with respect to expertise. Meanwhile, to learn more about the attention to visuomotor cues during segmentation and interactions with expertise, non-periodical movements of a single limb could be tracked with a 3D marker based system instead of video-based tracking. Moreover, segmentation of highly structured actions such as those employed here could be compared to less structured action sequences from Tai-Chi or contemporary dance.

### Summary

To summarize, the group of experts agreed on more boundaries than the novices. Taekwondo style and sequence complexity did not significantly modulate this effect. The exact superimposition of the experts’ responses in particular bins points to the use of predictive mechanisms based on sensorimotor representations. Prediction on the basis of semantic knowledge might have supported the selection of boundaries. Further, the overlap of the agreed boundaries in the expert group with those of the expert referee suggests that semantic representations and normative aspects were used which Taekwondoists but not novices were familiar with. Due to some limitations, the results can only provide a tentative insight into the content of shared representations.

## Data Availability Statement

The raw data supporting the conclusions of this article will be made available by the authors, without undue reservation.

## Ethics Statement

The studies involving human participants were reviewed and approved by the Ethics Committee of Tokyo Metropolitan University (reference number H28-69). The participants provided their written informed consent to participate in this study. Written informed consent was obtained from the individual(s) for the publication of any potentially identifiable images or data included in this article.

## Author Contributions

WS, JH, and MI conceived the study. VK and WS designed the stimuli and procedure. VK, WS, and MI collected and analyzed the data. RB contributed analyses of movement kinematics. JH provided scientific advice and commented the manuscript. WS wrote the manuscript. All authors contributed to the article and approved the submitted version.

## Conflict of Interest

The authors declare that the research was conducted in the absence of any commercial or financial relationships that could be construed as a potential conflict of interest.

## Publisher’s Note

All claims expressed in this article are solely those of the authors and do not necessarily represent those of their affiliated organizations, or those of the publisher, the editors and the reviewers. Any product that may be evaluated in this article, or claim that may be made by its manufacturer, is not guaranteed or endorsed by the publisher.

## Supplementary Material

Supplementary Videos To associate the agreed boundaries with the action sequence dynamically, we animated a mark which moves through the afv-graphs in synchrony with the movement video. In this way, the relation between agreed boundaries and the movement sequence might be more directly observable. For each video frame, the afv-graphs indicate how many participants marked a boundary. Peaks show that a high number of participants placed a mark, i.e., a boundary in the action sequence indicating that at the respective moment, a meaningful unit ends and a new one begins. The same videos as used in the movement analysis (Figure 5) are provided. The four videos show ITF Tul #5 and WT Poomsae #8, each segmented by experts (*n* = 24) and novices (*n* = 28). The videos display optimally VLC media player software (freeware).
