# Supplementary material for: Shared Representations in Athletes: Segmenting Action Sequences From Taekwondo Reveals Implicit Agreement
Source: Front Psychol. 2021 Nov 22;12:733896. doi: 10.3389/fpsyg.2021.733896 (PMC8645601; doi:10.3389/fpsyg.2021.733896)

Supplementary Figure 1 (S1).
Overlay between responses of T4 and added frame values (afv) of experts and controls.
Comparisons for all videos.

For each single video a comparison of the responses of an experienced referee (T4) with the added frame values (afv) of the expert group and the control group is shown.

Each of the 6 figures on each sheet (S1 A – D) shows the results of the action segmentation task (afv) for a single video. Each sheet contains either ITF (S1 A & S1 B) or WT (S1 C & S1 D) forms segmented by the experts or the control group. The order of the figures corresponds to the augmenting complexity of the sequences. The left upper panel shows the less complex sequence and the lower right the most difficult one.

The added frame values (afv) represent the number of participants responding within each bin (i.e., one second prior to each video-frame). Responses of the two blocks are summarized, thus, the maximal afv achievable is double the participants in each group (expert group without T4). Red lines represent the boundaries of T4 in block 1 and dashed black lines are boundaries from block 2. The exact times of the responses of T4 are at the line marking the end of the 1 sec bin during which they are counted.

Supplementary Figure 1 A. ITF – afv of experts compared to T4

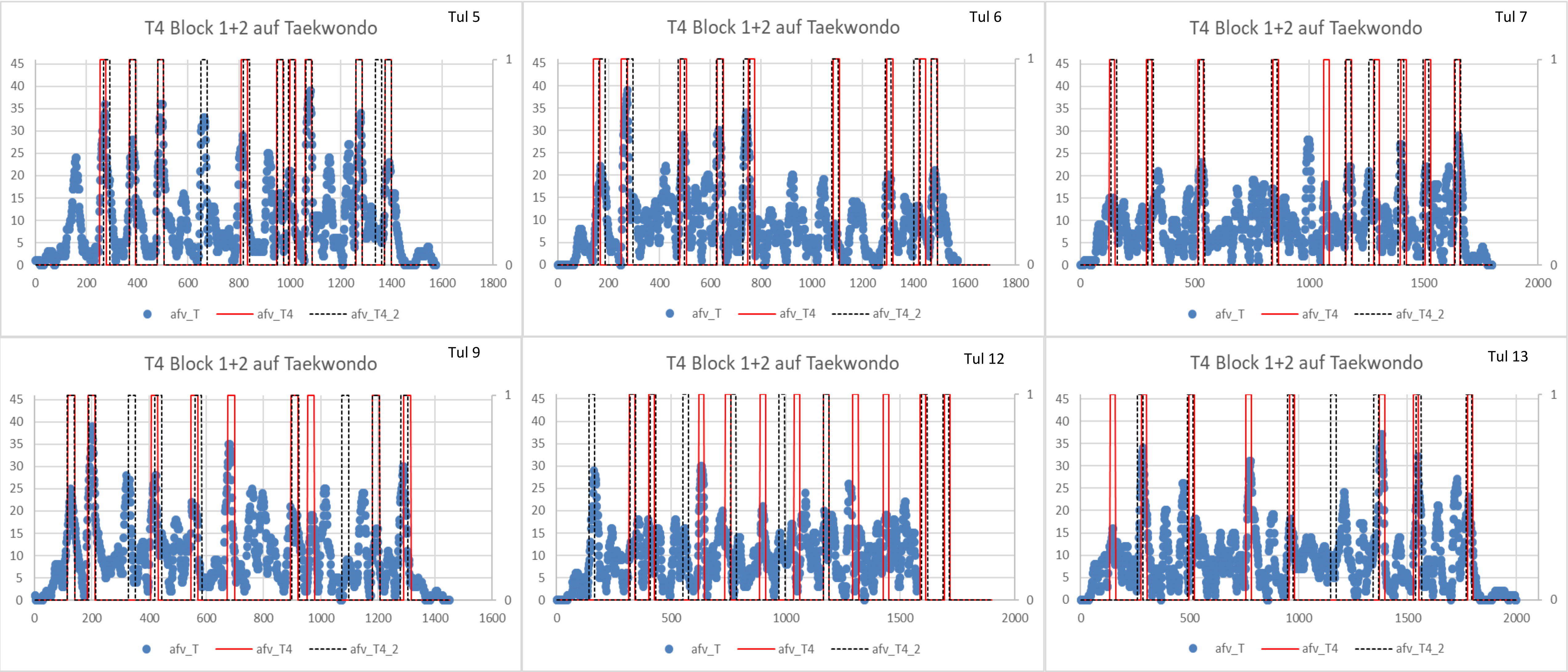

Supplementary Figure 1 B. ITF – afv of controls compared to T4

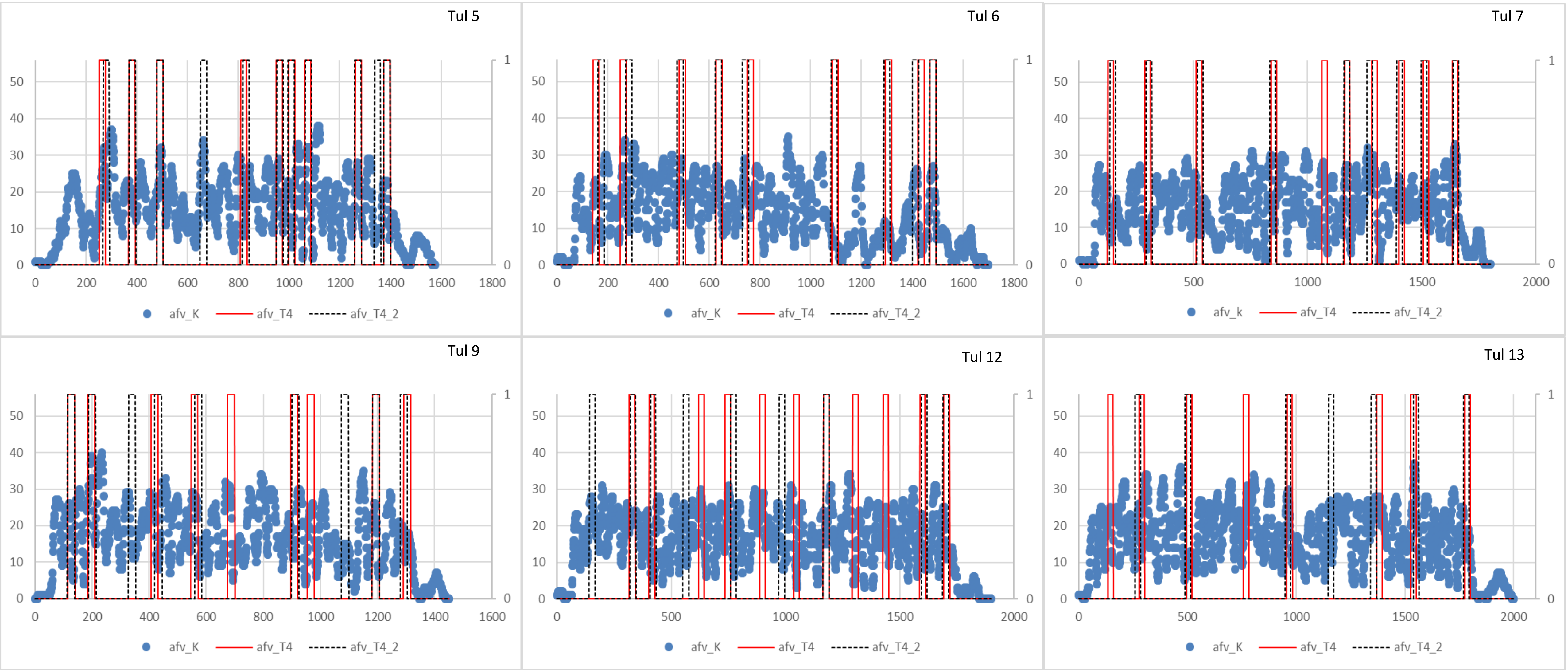

Supplementary Figure 1 C. WT – afv of experts compared to T4

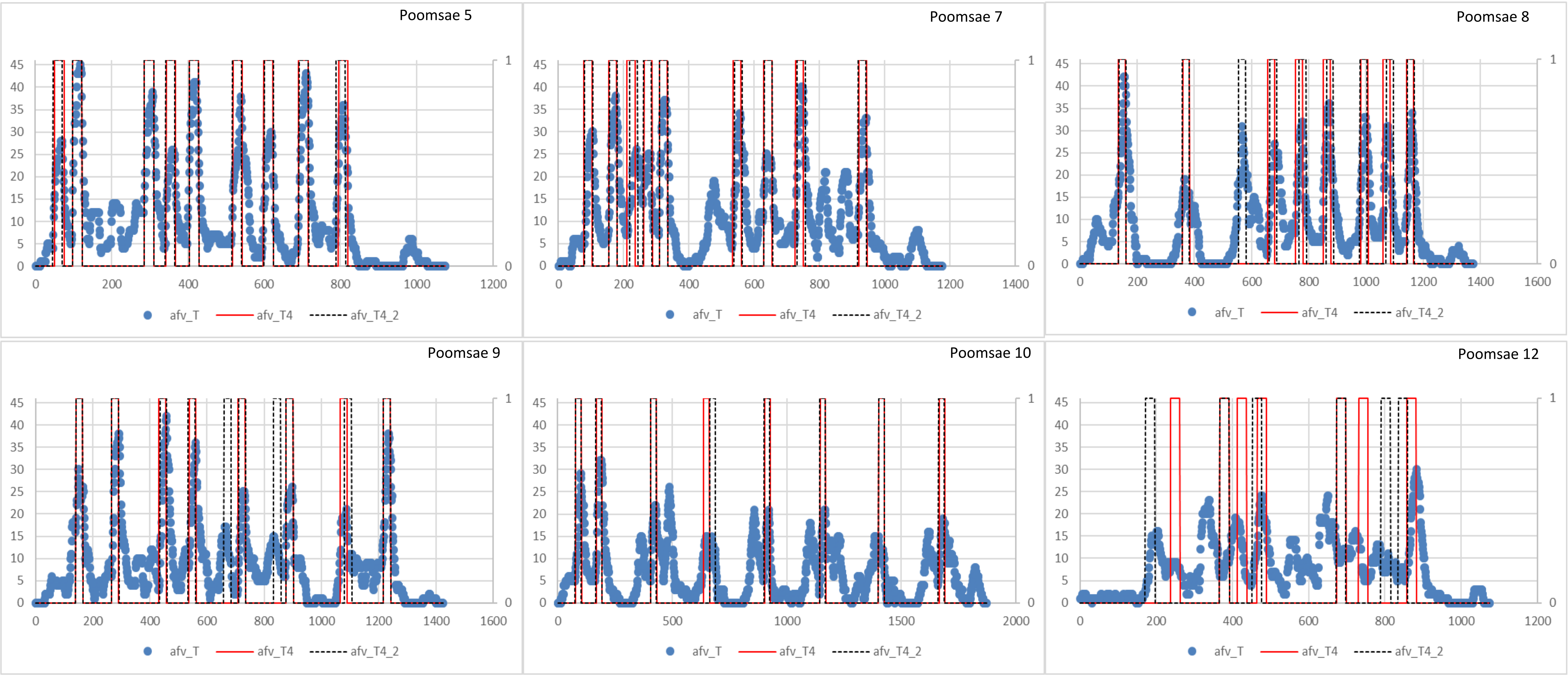

Supplementary Figure 1 D. WT – afv of controls compared to T4

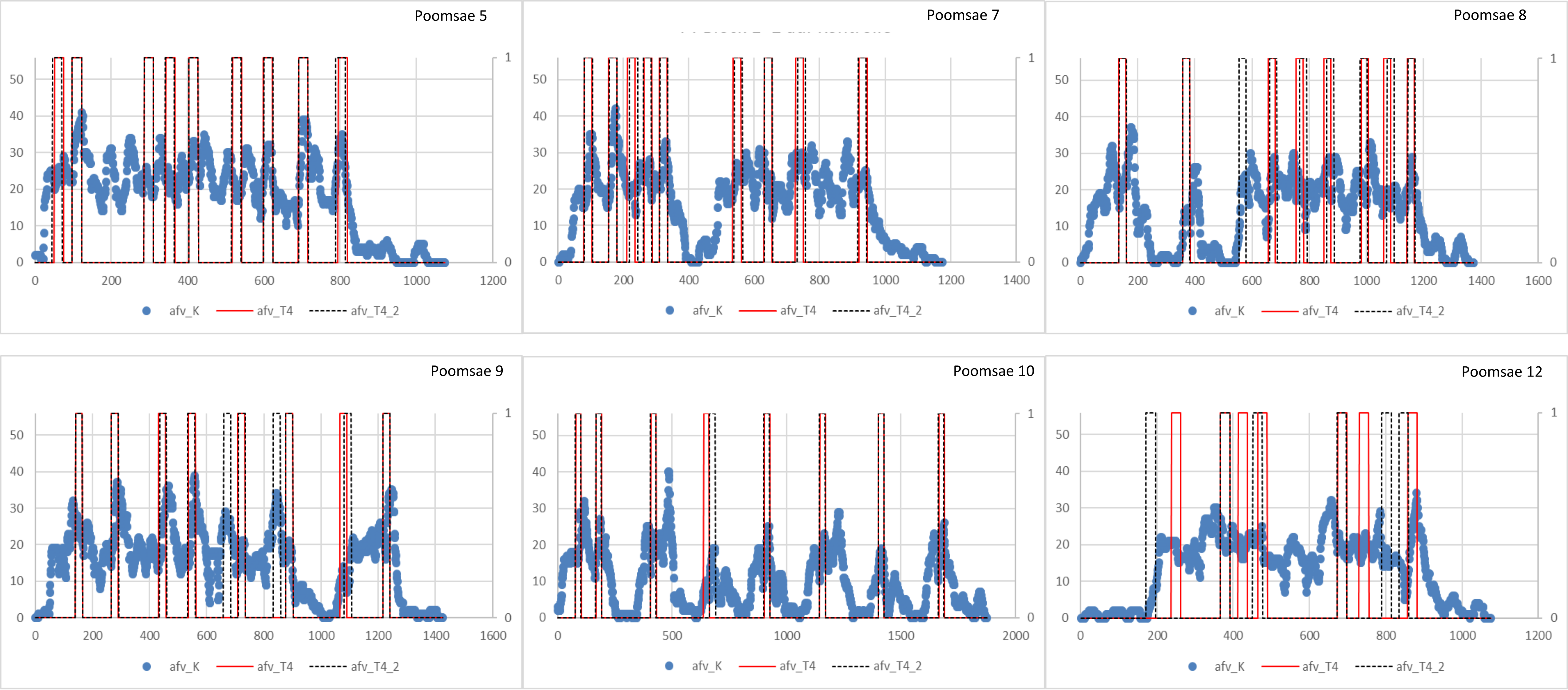

Supplement: Supplementary file 5 [file Data_Sheet_1.PDF]
