# Supplementary material for: Shared Representations in Athletes: Segmenting Action Sequences From Taekwondo Reveals Implicit Agreement
Source: Front Psychol. 2021 Nov 22;12:733896. doi: 10.3389/fpsyg.2021.733896 (PMC8645601; doi:10.3389/fpsyg.2021.733896)
Supplement: Supplementary file 6 [file Data_Sheet_2.PDF]

Supplementary Material

1 Supplementary Videos

To associate the agreed boundaries with the action sequence dynamically, we animated a mark which moves through the afv-graphs in synchrony with the movement video. In this way, the relation between agreed boundaries and the movement sequence might be more directly observable.

For each video frame, the afv-graphs indicate how many participants marked a boundary. Peaks show that a high number of participants placed a mark, i.e., a boundary in the action sequence indicating that at the respective moment, a meaningful unit ends and a new one begins. The same videos as used in the movement analysis (Fig. 6) are provided. The four videos show ITF Tul #6 and #13, each segmented by experts (n = 24) and novices (n = 28).

The videos display optimally VLC media player software (freeware).
